# Envelope Following Response to 440 Hz Carrier Chirp-Modulated Tones Show Clinically Relevant Changes in Schizophrenia

**DOI:** 10.3390/brainsci11010022

**Published:** 2020-12-27

**Authors:** Inga Griskova-Bulanova, Aleksandras Voicikas, Kastytis Dapsys, Sigita Melynyte, Sergejus Andruskevicius, Evaldas Pipinis

**Affiliations:** 1 Institute of Biosciences, Life Sciences Centre, Vilnius University, Saulėtekio av. 7, LT-10257 Vilnius, Lithuania; kastytis.dapsys@rvpl.lt (K.D.); sigita.melynyte@gf.vu.lt (S.M.); evaldas.pipinis@gmc.vu.lt (E.P.); 2 Vilnius Republican Psychiatric Hospital, Parko str. 21, LT-11205 Vilnius, Lithuania; aleksandras.voicikas@gmc.vu.lt (A.V.); sergejus.andruskevicius@rvpl.lt (S.A.); 3 Institute of Psychology, Mykolas Romeris University, Ateities str. 20, LT-08303 Vilnius, Lithuania

**Keywords:** auditory steady-state response, biomarker, envelope following response, gamma, schizophrenia

## Abstract

The 40 Hz auditory steady-state response (ASSR) impairment is suggested as an electrophysiological biomarker of schizophrenia; however, existing data also points to the deficiency of low and high frequency ASSR responses. In order to obtain the full picture of potential impairment in schizophrenia, it is important to test responses at different frequencies. The current study aims to evaluate a wide frequency range (1–120 Hz) in response to brief low-frequency carrier chirp-modulated tones in a group of patients with schizophrenia. The EEG-derived envelope following responses (EFRs) were obtained in a group of male patients with schizophrenia (N = 18) and matched controls (N = 18). While subjects were watching silent movies, 440 Hz carrier chirp-modulated at 1–120 Hz tones were presented. Phase-locking index and evoked amplitude in response to stimulation were assessed and compared on point-to-point basis. The peak frequency of the low gamma response was estimated. Measures were correlated with psychopathology—positive, negative, total scores of the Positive and Negative Syndrome Scale (PANSS), and hallucination subscale scores. In comparison to controls, patients showed (1) reduced power of theta-beta (4–18 Hz) responses, (2) intact but slower low gamma (30–60 Hz), and (3) reduced high gamma (95–120 Hz) responses. No correlation survived the Bonferroni correction, but a sign of positive association between low gamma phase-locking and the prevalence of hallucinations, and a sign of negative association between high gamma phase-locking and the total PANSS scores were observed. Brain networks showed impaired capabilities to generate EFRs at different frequencies in schizophrenia; moreover, even when responses of patients did not significantly differ from controls on the group level, they still showed potentially clinically relevant variability.

## 1. Introduction

In recent years, an immense interest for easily assessable brain signatures that can be used to diagnose or predict the outcome of psychiatric disorders has emerged. Electroencephalography (EEG), being a cheap and non-invasive technique, has been widely utilized for this purpose. The participation of cortical regions during various information processing steps translates into the synchronization of rhythmic neural activity at different frequencies [1] that are affected by disease [2] and can be registered from the scalp using EEG. Auditory steady-state responses (ASSRs) allow testing the ability of thalamo-cortical and local cortical circuits to produce and maintain synchronous activity at certain frequencies as a response to repetitive external stimulation [3,4]. The research in psychiatric populations is highly biased toward the assessment of responses in the 30–50 Hz range, and the stimulation parameters used for ASSRs are rather uniform. The 40 Hz ASSR has been proposed as a potential biomarker for schizophrenia [5,6]. However, it has been shown that the impairment of responses within the lower [7,8] and higher [9,10] frequency ranges in patients with schizophrenia also exists. Moreover, responses at different frequencies are generated by different sources, including thalamo-cortical pathways, sensory cortical, and deep cortical structures for low-frequency ASSRs (4–20 Hz) [11,12,13], local superficial networks for low-gamma range ASSRs (30–50 Hz) [14], and subcortical sources for 80 Hz ASSRs [15,16,17]. Thus, in order to obtain the full picture of potential impairment in the case of neuropsychiatric disorders, it is important to test the ability to produce and maintain synchronous activity at different frequencies [18].

However, long total stimulation periods are necessary to collect the responses to multiple stimulation frequencies; this makes experiments challenging for participants. Artieda et al. (2004) tested EEG responses to chirp-modulated tones that evoked envelope following responses (EFRs) [19]. EFR stands for a steady-state evoked response which follows the envelope of a stimulating waveform that covers a wide range of stimulation frequencies simultaneously [20]. This approach substantially shortened the data collection time in comparison to previous reports [21]. In this way, brief chirp stimulation allows efficient testing of responses to the desired frequency range, thus being advantageous in clinical settings. The potential to use chirp-modulated sound in schizophrenia research was shown by Alegre et al. (2017) [9]; however, the abovementioned study focused on the low (30–60 Hz) and high (90–110 Hz) gamma components only. Moreover, authors used a high frequency carrier (1200 Hz) that has been perceived as less pleasant by the subjects [22,23] and two-second length tones. Recently, a modified version of chirp stimulation with brief (500 ms) low frequency carrier (440 Hz) chirp-modulated at 1–120 Hz tones was introduced [24]. The sounds were perceived as neutrally pleasant and neutrally arousing, and EEG responses to the sounds were not affected by attentional manipulations [24]. Presumably, the proposed settings might be particularly suitable for patients with schizophrenia, who (1) frequently experience increased perceptual sensitivity to auditory stimuli [25,26] and (2) encounter problems in controlling attentional focus [27,28]. Moreover, proposed stimulation settings and point-by-point analysis of response revealed a specific impairment of low gamma activity in patients with disorders of consciousness [29].

The current study aims to evaluate a 2–120 Hz frequency range responses to brief chirp-modulated 440 Hz carrier tones in a group of symptomatic patients with schizophrenia in order to test the feasibility of this type of stimulation for further exploration. We utilize the point-by-point analysis of the EEG data [29] that permits the detection of the frequency ranges where groups show significant differences without imposing limits on the definition of frequency bands in order to uncover the complex electrophysiological abnormalities that might be observed in schizophrenia. We expect to observe impaired low frequency responses and low gamma and high gamma responses, as shown by previous works using discrete single-frequency stimulation settings.

## 2. Materials and Methods

### 2.1. Subjects

Eighteen inpatient males from the Republican Vilnius Psychiatric Hospital (SZ, mean age 38 years, SD 14 years) and 18 healthy age-matched male volunteers (H, mean age, 42 years, SD 13 years) participated in the study. The enrolment of male participants allowed excluding the potential influence of hormonal fluctuations on ASSRs [30]. The exclusion criteria were the history of organic illnesses, head trauma, and alcohol/substance abuse (except tobacco). The study was approved by the Bioethics Committee of Vilnius Republican Psychiatric Hospital (2009-05-15 Nr. V6-2), and written informed consents were obtained.

Patients were diagnosed with paranoid schizophrenia (ICD-10, mean illness duration 13 years, SD 10 years). They were interviewed using the Positive and Negative Syndrome Scale (PANSS) [31], and at the time of recruitment, patients commonly received either a combination of haloperidol with atypical neuroleptics (*n* = 9, like olanzapine), or a combination of olanzapine and risperidone (*n* = 2), or olanzapine (*n* = 4), quetiapine (*n* = 1), clozapine (*n* = 1), or haloperidol (*n* = 1) alone. Additionally, 14 patients received benzodiazepines (mainly diazepam or lorazepam). Table 1 shows the demographic and psychological characteristics of the schizophrenia patients (SZ) and healthy (H) control groups.

### 2.2. Stimulation

**Figure 1:**
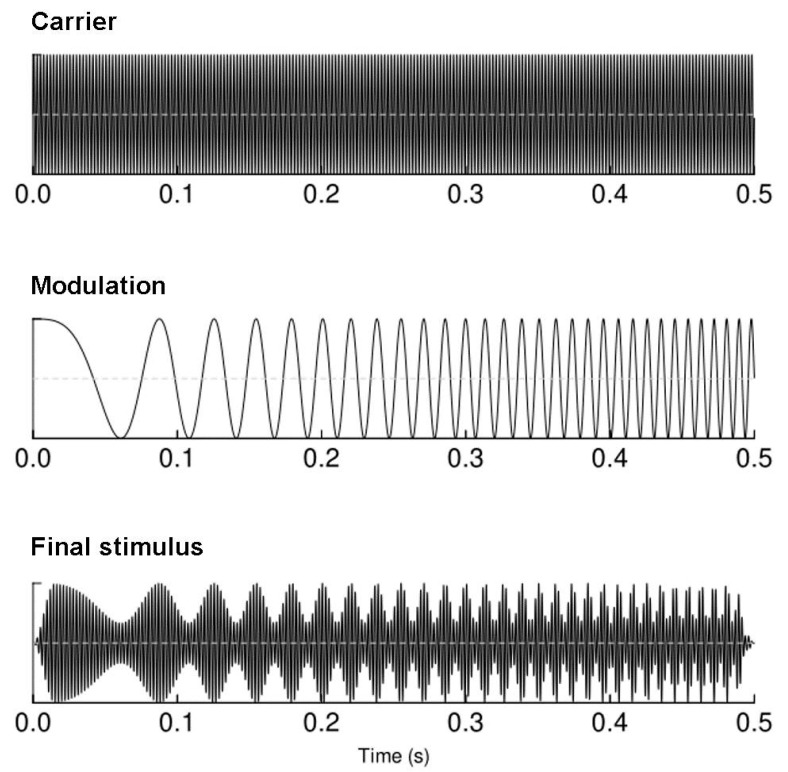
A schematic representation of the stimulus used to elicit envelope following responses.

The auditory chirp stimuli were designed in the MATLAB 2014 environment (The MathWorks Inc., Natick, MA, USA). The sounds were created as 440 Hz tones linearly modulated in the frequency window of 1 to 120 Hz (increasing modulation frequency); the modulation lasted 500 ms with 15 ms onset/offset linear ramps to avoid clicks [24]. The schematic representation of the stimulus is plotted in (Figure 1). The binaural presentation of sounds at 60 dBA (the relative loudness of sounds in air as perceived by the human ear) was performed through Beyer dynamic DT-1350 headphones (Beyerdynamic GmbH&Co, Heilbronn, Germany), while study participants watched a silent documentary movie on a screen in front of them [32]. Chirp stimuli were presented 450 times with ISIs set at 700–1000 ms.

### 2.3. EEG Recording

Continuous EEG was recorded with a Galileo Mizar Sirius computerized electroencephalogram system (EBNeuro, Florence, Italy). The ground electrode was attached at Fpz location, and earlobe electrodes served as the recording reference. Impedance was kept below 20 kΩ, and the sampling rate was set at 512 Hz. EEG from nine Ag/AgCl electrodes (F3, Fz, F4, C3, Cz, C4, P3, Pz, P4) were subjected to further analysis [32,33,34].

### 2.4. EEG Processing

The off-line data pre-processing and analysis was performed with custom written scripts for MATLAB (MATLAB 2010) using functions from EEGLAB [35], Fieldtrip [36], and ERPWAVELAB [37]. Raw EEG data were filtered with 1 Hz high-pass filter (EEGLAB, FIR). The CleanLine plugin for EEGLAB was employed to remove power-line noise by applying multi-tapering and Thomas F-statistics. Epochs were created starting –500 ms before the stimulus onset until 1000 ms post-stimulus and were re-referenced to the average reference. The excessively noisy epochs were determined by visual inspection and rejected by the researcher blind to the subject’s state. On average, 93% ± 7% for the control and 91% ± 6% for the patient groups of the epochs was left after the data cleaning. Data were baseline-corrected to the mean of the pre-stimulus period. A wavelet transformation was performed using complex Morlet wavelet from the Matlab© Wavelet Toolbox. The frequencies represented were from 1 to 120 Hz in 1 Hz intervals between each frequency. The phase-locking index (PLI, measuring the phase consistency of the response over epochs) and evoked amplitude (EA, corresponding to the wavelet-transformed evoked potential and representing the phase-aligned amplitude measure) were calculated [37]. Both PLI and EA were baseline-corrected by subtracting the mean activity of the prestimulus period (starting −400 ms before the stimulus onset until 0 ms) separately for each frequency. Chirp-EFRs were analyzed as the average of Fz and Cz electrodes that showed the most consistent activity for both subject groups; these locations are in line with a fronto-central topography of EFRs observed by Artrieda et al. (2004) and results of our previous work showing the EFR to be strongest at the fronto-central electrodes [20,24,29].

### 2.5. Data Analysis

**Figure 2:**
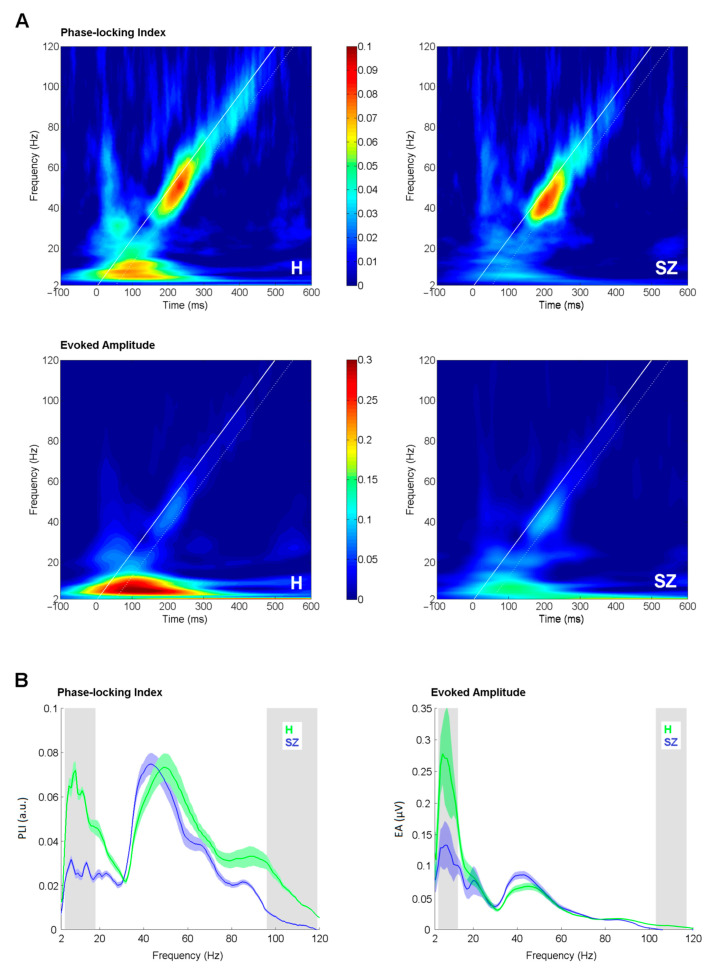
Comparison of envelope following response (EFR) measures between groups. (**A**) Time-frequency plots of phase-locking index (upper panel) and evoked amplitude (lower panel) in healthy controls (H) and schizophrenia patients (SZ) as an average of Fz and Cz electrodes. White bold line corresponds to the stimulation timing; white dashed line denotes +50 ms window of the analysis. (**B**) Phase-locking index and evoked amplitude curves as an average of Fz and Cz electrodes. The shaded color areas around the curve represent standard errors. The significantly different frequency windows, as assessed by the bootstrap, are marked by grey shaded areas. Green stands for healthy controls (H); blue stands for schizophrenia patients (SZ).

Phase-locking index and evoked amplitude curves (Figure 2B) were derived by averaging EFR in the time window of +50 ms separately for each frequency point starting from 2 to 120 Hz in 1 Hz step. The starting time point for averaging was set at the initiation time point for each stimulation frequency. The white diagonal line in (Figure 2A) represents the time-course of stimulation, and the white dashed line delineates the window for the analysis. The extracted PLI/EA curves were further subjected to a bootstrap t-test (1000 iterations, *p* < 0.05) to perform a point-to-point comparison between study groups. 95% bootstrapped confidence intervals were used to assess significance. Although the bootstrap test is less conservative option for solving the multiple comparison problem than the Bonferroni test, it is robust, stable [38] and widely used in EEG analysis [39,40,41]. Additionally, to allow for comparison to earlier reports, Pearson correlation was assessed between PLI/EA curves and positive, negative, total PANSS and hallucination subscale scores. A Bonferroni adjusted α-level of 0.0001 (0.05/480) was regarded as significant.

The peak frequencies of the low gamma response (30–60 Hz range, defined as the frequency point at which the strongest response was observed) were estimated and compared using independent sample t-test, as implemented in SPSSv20 (SPSS Inc., Chicago, IL, USA).

## 3. Results

The time-frequency plots of PLI and EA in controls and patients are depicted in (Figure 2A). PLI and EA curves from the control and patient groups are presented in (Figure 2B). For PLIs, the peak at the low gamma range (30–60 Hz) was clearly distinguishable. The peak frequency in the patient group (44 Hz, SD 7) was significantly slower (t = 2.096, df = 34, *p* = 0.04), as compared to the controls (49 Hz, SD 8).

The significant difference in the degree of phase-locking between controls and patients was observed in two frequency windows: from 4 to 18 Hz (0.001 < *p* < 0.045, 0.50 < Hedges’ g < 1.23) and from 96 to 119 Hz (0.009 < *p* < 0.046, 0.41 < Hedges’ g < 0.73). For EAs, the significant difference between patients and controls emerged from 4 to 13 Hz (0.003 < *p* < 0.041, 0.50 < Hedges’ g < 0.87) and from 103 to 117 Hz (0.001 < *p* < 0.032, 0.24 < Hedges’ g < 0.50). Lower PLI and EA values were observed in the SZ group in both frequency windows.

**Figure 3:**
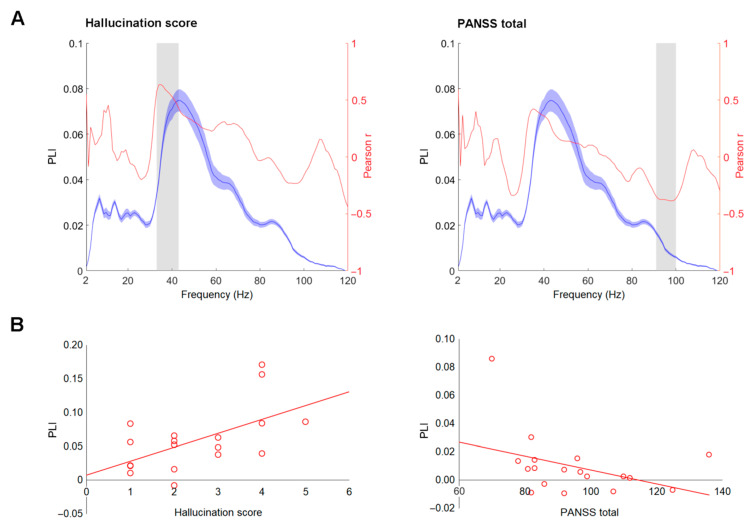
Correlation between envelope following responses (EFR) measures and clinical variables. (**A**) Phase-locking index (PLI) curves (blue) and Pearson r values (red) between PLI and hallucination scores and Positive and Negative Syndrome Scale (PANSS) total scores in schizophrenia group. The shaded blue areas around the curve represent standard deviations. The frequency windows where significant correlation was observed, as assessed by the bootstrap, are marked by grey shaded areas. (**B**) Scatterplots of PLI values averaged within the significant window (depicted in part A) against hallucination and PANSS total scores.

The associations between PLI/EA and clinical variables—positive, negative, total PANSS scores, and hallucination subscale score—were assessed in the patient group. None were significant at a Bonferroni adjusted α-level of 0.0001 (0.05/480). However, we report the associations with uncorrected *p* values (*p* < 0.05) for exploratory and comparative purposes. The PLI values in the 32–43 Hz range showed a sign of positive correlation to the hallucination score (0.403 < r < 0.636, 0.003 < *p* < 0.044). The total PANSS scores showed a sign of negative relation to PLI values in the 91–100 Hz frequency window (−0.386 < r < −0.347, 0.028 < *p* < 0.049) and to EA values in the 95–101 Hz frequency window (−0.378 < r < −0.352, 0.030 < *p* < 0.048). For visualization purposes, the plots showing the course of Pearson r values for association between PLIs and hallucination scores, PLIs and total PANSS scores (Figure 3A), and corresponding scatterplots are depicted in Figure 3B.

## 4. Discussion

We aimed to evaluate the dysfunctional ability to synchronize at a broad range of frequencies in schizophrenia. We employed brief (500 ms) chirp-modulated at 1–120 Hz low carrier frequency tones, using the benefit of this type of stimulation—an assessment of a wide frequency range in a short stimulation session. Additionally, we employed point-to-point comparison of EFRs, avoiding focusing on predefined EEG band limits. This resulted in several important observations. First, an impaired synchronization and strength of responses at theta-beta (4–18 Hz) and high gamma (95–120 Hz) ranges was revealed in patients. Second, the response at low gamma (30–50 Hz) range was preserved in the schizophrenia group, but the peak of individual gamma activity in the patient sample was slowed in comparison to the control group.

To our knowledge, this is the first study where a wide frequency range is evaluated in a sample of symptomatic inpatients with schizophrenia without prior constrains on the frequency windows of interest. Previous sparse studies employing chirps [9,24] focused mostly on pre-defined low and high gamma activity in response to the auditory stimulation. The point-to-point comparison of extracted phase-locking and evoked amplitude curves spanning all stimulation frequency range is beneficial, as it allows obtaining the full picture of potential impairment. This is particularly important, as (1) responses at different frequency ranges are potentially generated by different sources, and (2) the altered synchronization properties in patients were previously shown, not only using ASSRs at low gamma range (30–40 Hz), but also lower (2–8 Hz) and higher (80 Hz) frequencies. The assessment of all frequency ranges separately would result in a long stimulation duration. The proposed chirp-modulated low carrier frequency tones allowed efficient testing of responses to the wide frequency range with one stimulus in patients with schizophrenia, who could potentially experience increased perceptual sensitivity to auditory stimuli and encounter problems with attentional focus.

Until now, the majority of studies in clinical populations aimed at the evaluation of 40 Hz ASSRs. Impaired responses within the low gamma range (30–40 Hz) were reported in schizophrenia (for review refer to Thuné et al., 2016 [6]), affective disorders [42,43], and autism [44]. Respectively, a reduction of strength and phase-locking of ASSRs in the 30–60 Hz range in the patient group was expected. However, no significant differences in PLI and EA of the low gamma window were found between controls and patients. The unimpaired [45], or even increased [8,46], 40 Hz ASSRs in patients with schizophrenia were reported by several authors before and were related to the effect of medication status [45], older age of subjects [7] or recording conditions [47]. In line with our results, Alegre et al. (2017) have previously shown impaired low gamma (30–50 Hz) responses in untreated patients but not in medicated patients [9]. Our sample was characterized by expressed symptoms and all subjects received antipsychotic medication that could have normalized activity in the 30–50 Hz range. Additionally, although no effect of distraction on responses to chirp stimulation with similar to current settings in healthy subjects was observed [24], the exact effect of distractive tasks (e.g., silent documentary watching) on EFRs in patients is not known. Some evidence suggests that ASSRs in the low gamma range may be modulated by recording conditions in patients [33,48], thus this effect cannot be fully withdrawn. Finally, no correlation between age and low gamma response was observed either in patients or in controls, though decline of 40 Hz ASSRs with age was reported before [34,49].

Despite the lack of differences between patients and controls in the synchronization and strength of the response within the 30–60 Hz range, two important features of the low gamma responses in the patient group deserve being discussed. First, a sign of a positive relationship between PLI values in the 32–43 Hz range and hallucination scores in patient group was observed, although it did not survive the Bonferroni correction. However, this observation corresponds to the earlier report by Spencer et al. (2009) [50] on conventional 40 Hz ASSRs, where a positive correlation was shown between parameters of 40 Hz ASSRs and hallucination prevalence. This also demonstrates that even when not different from the healthy subjects on the group level, responses originating in the local superficial networks [14] and reflecting short-distance synchronization properties [51] still potentially show clinically relevant variability in patients. Although the correlation performed should be regarded as exploratory, and the relatively small sample size does not allow a firm conclusion, the coincidence in findings with previous reports suggests the importance of this observation and highlights the aspect that should be evaluated in future studies. Secondly, the shift of the low gamma peak towards lower frequencies in the patient group was revealed. In the control group, the PLIs peaked at around 49 Hz and the response was maximal at around 44 Hz in patient group. The shift towards lower frequencies in schizophrenia was reported before [47,52,53,54] and linked to the prevalence of negative symptomatology [47,53] and higher self-disorder scores [55]. Although, in the current patient sample no significant correlation between the peak frequency in the low gamma range and any of the psychopathology scales was found, the observed slowing deserves further attention, as it might indicate impaired dynamic interactions between multiple brain regions [53].

The 40 Hz ASSR impairment is suggested as an electrophysiological biomarker of the disorder [6]; nevertheless, existing data also points to the deficiency of lower [7,8] and higher frequency [9,10] responses in schizophrenia. Our results are in compliance with previous findings of attenuated low frequency auditory evoked responses (2–8 Hz) obtained with conventional ASSR settings in patients [7,8,56,57]. The responses at the 4–18 Hz range are considered to originate in the thalamo-cortical pathways and in the sensory cortical and deep cortical structures [11,12,13] and presumably reflect long-distance synchronization abilities [51]. It is plausible that the large synchronous response in the low frequency range (Figure 1) could have been produced by the onset of the auditory stimulus; thus, the impaired response seen in our patient sample likely reflects the robust auditory encoding abnormalities observed in schizophrenia [58,59,60] that are evident at both oscillatory and event-related potential component levels [61]. The potential of brief chirp stimulation to reveal impaired responses in low frequency range also supports recent findings by Edgar et al. (2018) [7], who proposed that alterations of low frequency responses to auditory stimulation in schizophrenia are more stable than impairments of 40 Hz ASSR. Presumably, this effect could be related to the involvement of a wider range of affected networks tested with responses at low frequencies [11,12,13]. Importantly, the findings of this work expand the relevant frequency range, pointing to the necessity of more studies addressing the low frequencies, including the theta, alpha, and early beta ranges.

Additionally, the reduction of the PLI and EA measures of ASSRs at higher gamma frequencies (90–120 Hz) in the patient group was evident. The contribution of high gamma-band activity towards the pathophysiology of schizophrenia is still unclear. The high frequency ASSRs are considered to be predominantly generated by the subcortical sources in the brainstem [15], and thalamus [16,17]; thus the impairment of the activity in this frequency range may indicate impaired functioning of networks at the subcortical level. This assumption is supported by both functional and anatomical alterations in subcortical structures reported in schizophrenia. Nopoulos et al. (2001) described changed morphology of the mid-brain to be related to psychotic symptoms and medication in SZ, with greater exposure being associated with greater morphologic impairments [62]. Similarly, the dysfunctional pattern in the thalamic functional connectivity of patients with schizophrenia was related to psychopathology in the study by Gong et al. (2019) [63]. Furthermore, several early reports showed impaired auditory brain-stem responses in patients with schizophrenia and its association to clinical symptom severity [64,65,66]. Importantly, the clinical relevance of ASSRs at higher frequencies was suggested by some reports: the inability to generate a high gamma band (80 Hz) response correlated to negative symptoms in the report by Hamm et al. (2011) [57] and to the severity of global hallucinatory experiences, as shown by Tsuchimoto et al. (2011) [10]. In the current study, a sign of a negative correlation (though not surviving the Bonferroni correction) between the PLI and EA values in the 91–101 Hz range and total PANSS scores was observed, signaling that impaired high gamma response may be related to the general level of psychopathology, and that stimulation with brief chirp-modulated tones is potentially capable to unravel this association.

Overall, our results support the notion that studying the full spectrum of gamma and low frequency oscillations may be critical for decoding the complex electrophysiological abnormalities observed in schizophrenia patients, as proposed by Moran and Hong (2011) [18]. However, several limitations of this report are worth mentioning. First of all, the small patient sample size prevented the detection of smaller effects. The observed correlations, due to the small sample size and a high number of tests did not survive the Bonferroni correction and should be treated with caution; however, they can be used as a guide for further systematic explorations. Secondly, our sample, though being homogenous in respect to diagnosis, was mostly on compound medications. Future studies should opt for patients on a more homogenous treatment regimen. Lastly, in order to keep the electrophysiological assessment as brief as possible, we did not employ single stimulation frequencies; however, it would be of great interest to compare results obtained with chirp stimulation to the outcomes of classical stimulation approaches, as to our knowledge, it has not been done in clinical schizophrenia samples before. Our earlier results in patients with disorders of consciousness are very promising in this respect, as the same associations were shown in response to both single frequency 40 Hz stimulation and to chirps similar to those used in the current study [29,67].

## 5. Conclusions

The results of the current study support the idea that brain networks in schizophrenia show impaired capabilities to generate responses to auditory stimulation at different frequencies: in comparison to healthy controls, patients showed (1) reduced theta-beta (4–18 Hz) responses, (2) intact but slower low gamma (30–60 Hz) responses that showed a sign of positive correlation to the prevalence of hallucinations, and (3) reduced high gamma (95–120 Hz) responses that showed a sign of negative correlation to the total PANSS scores. These alterations can be tested using brief low carrier frequency chirp-modulated tones that help capture various aspects of the impairment in short assessment sessions. The findings also demonstrate that even when not different from the healthy subjects on the group level, responses of patients are still showing clinically relevant variability.

## Figures and Tables

**Table 1:** Demographic and clinical characteristics of the study groups.

	H	SZ
Male/female, n	18/0	18/0
Age (years)	42 ± 13	38 ± 14
Smoking status (yes/no)	14/4	9/9
Onset age (years)		25.14 ± 12.45
Duration (years)		13.03 ± 9.76
Positive and negative syndrome scale:		
Positive		21.11 ± 5.43
Negative		28.50 ± 5.19
General		46.61 ± 10.53
Total		95.06 ± 17.31
Hallucinations		2.50 ± 1.29
Chlorpromazine equivalents (mg)		583.50 ± 217.03

## Data Availability

The data presented in this study are available on request from the corresponding author. The data are not publicly available due to privacy restrictions.
